# Supplementary figures and images for: A Vaccine Based on Asia1 Shamir of the Foot-and-Mouth Disease Virus Offers Low Levels of Protection to Pigs against Asia1/MOG/05, Circulating in East Asia
Source: Viruses. 2022 Aug 4;14(8):1726. doi: 10.3390/v14081726 (PMC9412984; doi:10.3390/v14081726)

## Slide 1
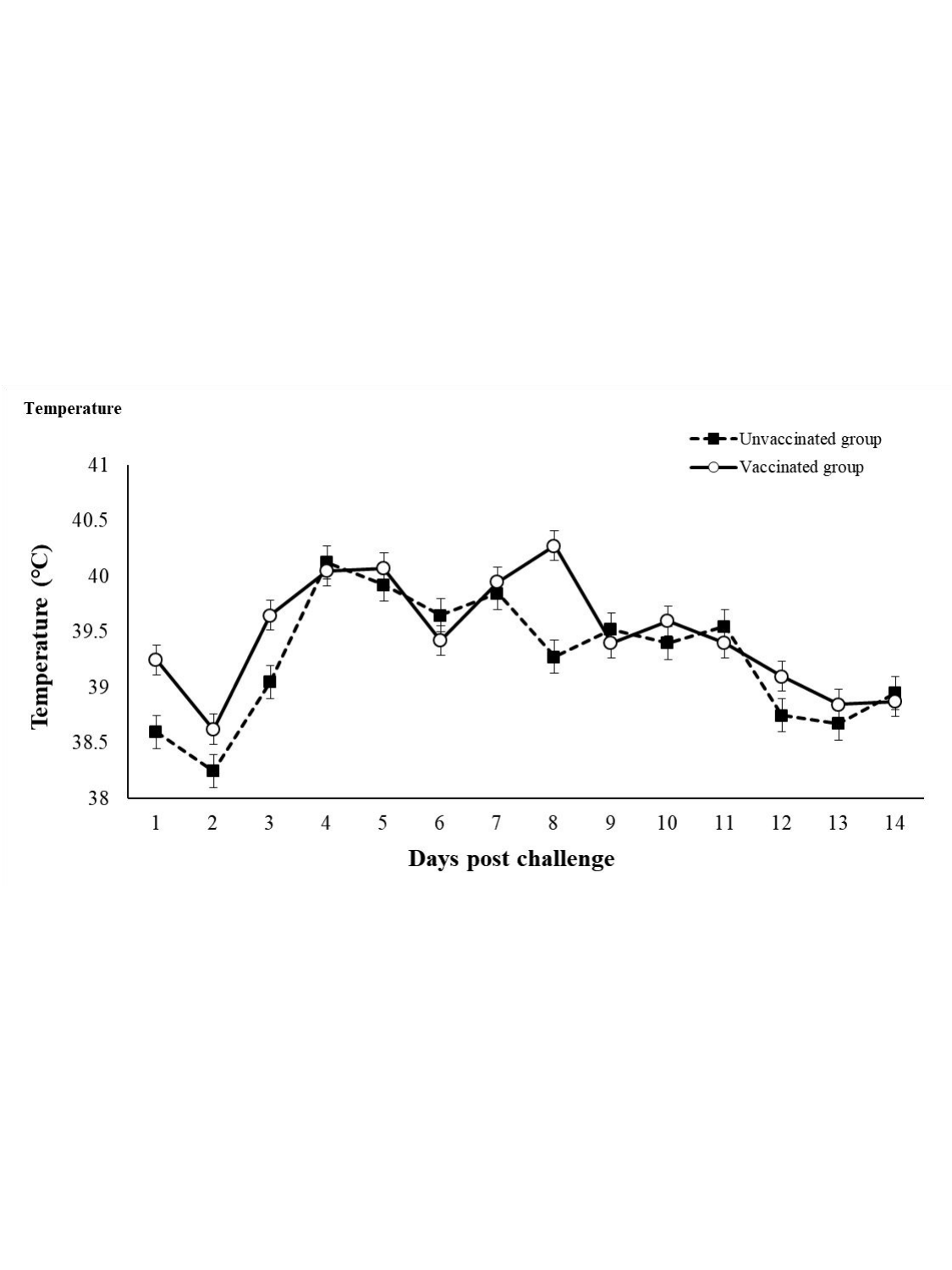

Supplement: Supplementary file 1 [file viruses-14-01726-s001.zip › viruses-1809419-supplementary.pptx]
